# Retrospective Evaluation of Submandibular Fossa Depth in Relation to Mandibular Canal and Bone Thickness: CBCT-based Study

**DOI:** 10.2174/0115734056386043250730170653

**Published:** 2025-08-21

**Authors:** Hasret Tanrıverdi Şahan, Mehmet Emin Doğan, Esin Akol Görgün

**Affiliations:** 1 Department of Dentomaxillofacial Radiology, Harran University, Faculty of Dentistry, Sanliurfa,, Turkey; 2 Department of Dentomaxillofacial Radiology, Adiyaman University, Faculty of Dentistry, Adiyaman, Turkey

**Keywords:** Submandibular fossa, Mandibular canal, Cortical bone, Cone-beam computed tomography, Buccal bone thickness, Lingual bone thickness

## Abstract

**Introduction:**

This study aimed to determine the depth of the SF, bone thicknesses in the buccal and lingual areas of the mandibular canal (MC), vertical positions of the SF and MC relative to each other, and the tooth level at which the deepest point of the SF was observed in the cross-sectional section.

**Methods:**

440 cone beam computed tomography (CBCT) images were retrospectively evaluated. The depth of the SF was determined. The buccal bone thickness (BBT) and lingual bone thickness (LBT) of the MC were measured, and the tooth alignment of the deepest point of the SF and the vertical position of the SF and MC relative to each other were determined.

**Results:**

In both jaws, SF depth Type I ratios were lower in males than in females, and SF depth Type III ratios were higher than in females. When the relationship between the vertical position of the MC and the region where the SF was deepest was examined, it was observed that the MC was in an inferior position in most patients.

**Discussion:**

In order to reduce the complication rate in the SF region, the relevant region should be analyzed in detail with CBCT before surgical procedures. The main limitation of our study is that the number of men and women was not equal.

**Conclusion:**

SF depth and BBT values in the right and left jaws were higher in males than in females. LBT was higher in females in the right jaw. As the depth of the SF increased, BBT and LBT values decreased.

## 1 INTRODUCTION

The SF, where the submandibular salivary gland is located, is an anatomical formation located in the mandibular posterior region, lingual to the corpus, between the inferior mylohyoid border and the inferior border of the mandible. The anteroposterior border of the SF is between the distal part of the mental foramen and the mesial part of the 3rd molar, but these borders are not clear [1-3]. Since the lingual of the mandibular posterior region is a dense region in terms of vascularization, including the sublingual and submental arteries, complications such as bleeding and infection may be observed in surgical procedures [4-6]. The complication rate will also increase in flapless operations and when the SF is deep [4, 6-8]. SF depth, which varies from person to person, affected implant placement in approximately 18.27% of cases when a bone height of 10 mm, *i.e*., a standard implant, was considered [9]. An SF depth greater than 2 mm increases the risk of complications that may occur during surgical procedures and implant placement in the relevant area [6].

In studies on the MC, where the inferior alveolar nerve, artery and vein are located [10] it has been determined that the location of the canal is closer to the lingual cortical plate in the molar region, closer to the buccal cortical plate in the premolar region and the 1st molar region, the canal is located in the middle of the buccal and lingual cortical bones [11]. Perforation of the canal may cause paresthesia in the jaw and corner of the mouth and devitalization of the teeth [6].

The structure of the bone in the relevant region and the relationship between the implant and the anatomical structures should be known before the placement of dental implants, which are frequently preferred in tooth deficiencies today. Thus, conditions such as bone perforation, infection, bone fracture, and implant loss can be prevented [6, 12, 13]. During implant surgery, complications such as bleeding, nerve damage, damage to the teeth adjacent to the area, and leakage of the implant into anatomical gaps may occur. Anatomical formations in the areas where the surgical procedure will be performed may increase the risk of complications that may occur [5, 14]. The mandibular posterior region, which is a risky region in terms of surgical procedures, should be carefully examined before surgical procedures due to anatomical formations such as the mandibular canal, submandibular fossa, submental and sublingual arteries located in this region [6, 12, 13].

Imaging methods such as periapical radiography, panoramic radiography, CBCT, and computed tomography (CT) can be used to evaluate the mandibular posterior region [1-3, 15]. Panoramic radiography has disadvantages such as image magnification and the inability to measure the buccolingual thickness of the bone [1, 4, 16, 17]. To prevent complications, CT and now CBCT imaging methods are used as the most appropriate techniques for evaluating the area to be treated before surgery [1, 4]. CBCT images can be preferred over CT in detailed analysis of hard tissues, implant planning, and reducing complications in surgical procedures; both by providing high-quality images and by using lower radiation doses compared to CT [5, 18].

This study aimed to determine the depth of SF in the examined CBCT images and the tooth alignment of the region where the deepest SF was observed. In addition, determining the bone thicknesses in the buccal and lingual areas of the MC and the vertical positions of the SF and MC relative to each other in the cross-sectional section where the deepest SF was observed were among our aims.

## 2 MATERIALS AND METHODS

This study was approved by the Non-Interventional Clinical Research Ethics Committee of Adiyaman University with the decision numbered 2024/2-4 dated 20/02/2024. It was conducted by the rules of the Declaration of Helsinki and has been prepared following the STROBE guidelines.

The G Power 3.1.9.4 package program was used for sample size calculation. In this study, the sample size was calculated using the Correlation Analysis Test solution to test whether there is a relationship between submandibular fossa depth and mandibular canal and bone thickness. Effect size, which is widely used in sample size calculations, needs to be calculated. ρ value reference ranges were suggested by Cohen for calculating effect size [19]. In addition, ρ value is interpreted as small, medium, and high effect size as 0.10, 0.30, and 0.50, respectively [19]. Therefore, when the correlation coefficient for sample calculation is 0.30 (Pearson correlation coefficient: ρ), the probability of error is 0.05, and the power of the study is accepted as 99%, it was seen that at least 195 individuals should be reached.

The intra-observer reliability of a test is evaluated by whether the same person or groups obtain similar results by repeating the same test. In this context, the submandibular fossa thickness, buccal thickness, and lingual thicknesses measured at different times on the right and left jaws of 25 individuals were compared. It was observed that the submandibular fossa thickness, buccal thickness, and lingual thicknesses measured at different times on the right jaws of 25 individuals were similar *(p>0,05).*

In this study, 440 cross-sectional images were obtained from 220 patients over the age of 18 who were admitted to the Department of Oral and Maxillofacial Radiology, Faculty of Dentistry Adiyaman University, between 2019 and 2022, and were retrospectively analyzed for various reasons. The images were captured using the Planmeca Pro Max 3D Mid Proface (Planmeca Oy, Helsinki, Finland). During irradiation, the patient stood with their head positioned so that the sagittal and vertical planes were perpendicular to the ground and the orbitomeatal plane was parallel to the ground. A single 360º rotation was performed during the exposure. The voxel size of the images obtained was 0.4 mm^3^, and the slice thickness was 0.40 mm. The images of the maxilla and mandible obtained from two consecutive exposures with a field of view (FOV) size of 16 x 9 cm were merged using the Romexis 4.6.2.R software program (Planmeca Oy, Helsinki, Finland). One mm cross-sectional images obtained from axial sections were evaluated retrospectively. Patients were divided into four age groups: 19-29 years, 30-39 years, 40-54 years, and 55 years and older. According to their dentition, mandibular first and second molars were included in the study. Patients with both first and second molars were classified as 'toothed', those without both teeth were classified as 'edentulous', and patients with one tooth but not the other were classified as 'monodentulous'.

Criteria for inclusion of images in the study: Images of individuals aged 18 and over, images in which the mandible is fully visualized, images in which the MC is clearly visible.

Criteria for exclusion of images from the study; Images where the mandible cannot be clearly viewed, CBCT images of individuals under the age of 18, CBCT images of individuals with severe bone loss for both hemi-mandibles, CBCT images with implants in the relevant region, CBCT images with impacted teeth in the relevant region, CBCT images with signs of oral surgery in the relevant region, CBCT images with pathology in the relevant region, CBCT images with fractures in the relevant region, CBCT images that are not of sufficient diagnostic quality, CBCT images where the MC cannot be clearly viewed.

The same observer, possessing at least three years of maxillofacial CBCT experience, examined the CBCT images and took the measurements. The parameters determined for measurement were as follows:

### 2.1 Depth of SF

**Fig. (1):**
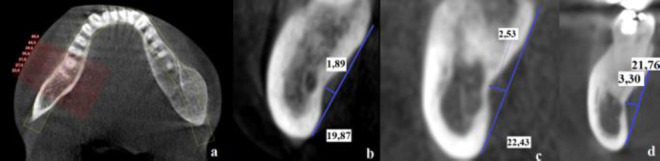
Images of the techniques used to measure SF depth in axial (**a**) and cross-sectional (type I (**b**), type II (**c**), type III (**d**) sections.

The section where the SF was observed to be the deepest was determined. In this section, the perpendicular distance drawn from the deepest point of the fossa to the tangent drawn to the upper and lower outermost points of the concavity was determined as the depth of the fossa. The deepest value of the SF was categorized into 3 different SF types based on the classification of Parnia *et al*. [6]. Type I: The group in which the depth of the fossa was up to 2 mm; type II: Group in which the depth of the fossa was between 2 and 3 mm; type III: Group in which the depth of the fossa was more than 3 mm (Fig. **1**).

### 2.2 Evaluation of the Relationship Between the Vertical Position of the Mandibular Canal and the Deepest Submandibular Fossa

**Fig. (2):**
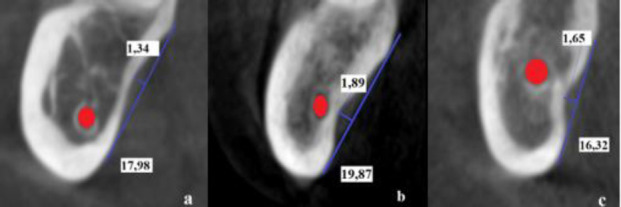
Cross-sectional sections showing the position of the MC relative to the deepest part of the SF inferiorly (**a**), parallel (**b**) and (**c**) superiorly.

In the section where the deepest fossa was measured, the position of the MC relative to the SF was classified as inferior, parallel, or superior. In the superior relationship, the vertical position of the MC was above the region where the SF was deepest. In a parallel relationship, the vertical position of the MC was adjacent to the deepest part of the SF. In the inferior relationship, the vertical position of the MC was below the region where the SF was deepest [13] (Fig. **2**).

### 2.3 Tooth Number

In patients with teeth, the tooth alignment of the deepest point of the SF was recorded.

### 2.4 Buccal Bone Thickness and Lingual Bone Thickness Measurement

**Fig. (3):**
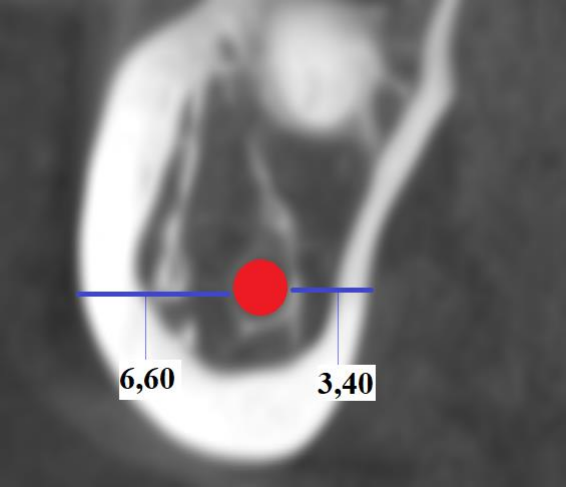
BBT and LBT images in cross-section.

In the section where the SF was deepest; the distance parallel to the horizontal plane from the midpoint of the buccal cortical border of the MC to the buccal cortical border was BBT, the distance parallel to the horizontal plane from the midpoint of the lingual cortical border of the MC to the lingual cortical border of the bone was considered as the LBT [20] (Fig. **3**).

### 2.5 Statistical Analysis

Data were analyzed with the Statistical Package for the Social Sciences (SPSS) 26.0 Statistics Package Program. The Chi-Square Test analyzed the distribution of the data. Independent Sample T-Test and One-Way ANOVA test were used to compare the means. Differences between groups were found with Post Hoc tests. Paired Sample t-test was used to compare the right and left jaw measurements and to test the reliability of observation. Pearson Correlation tests were used to examine the relationships between the gender, age, presence of teeth, and SF depth, BBT, and LBT parameters in the right and left jaws. In the whole study, significance levels were accepted as p<0.05.

## 3 RESULTS

In the Paired Sample T-test performed to evaluate the intra-observer agreement, the coefficient for the right jaw was 0.331, 0.194, and 0.052 for SF depth, BBT, and LBT, respectively; the coefficient for the left jaw was 0.416, 0.192, and 0.790 for SF depth, BBT, and LBT, respectively. When the gender distribution was analyzed in our study, 134 (60.9%) of the participants were female and 86 (39.1%) were male. When the age ranges were analyzed, 39.5% of the participants were between the ages of 19-29, 27.7% were between the ages of 30-39, 17.7% were between the ages of 40-54, and 15.0% were 55 years and over. The average age was 36.66 ± 13.94 (minimum 19, maximum 92).

The comparison of the findings regarding the anatomical structure of the right and left jaws and the dental regions examined according to the gender of the individuals is shown in Table **1**.

The distribution of SF depth types in the right and left jaws showed a significant difference according to the gender of the individuals (p<0.05). In both jaws, SF depth Type I ratios were lower in males than in females, and SF depth Type III ratios were higher than in females. A significant difference was observed between the mean values of SF depth, BBT, and LBT in the right jaw according to the gender of the individuals (p<0.05). While the mean values of SF depth and BBT were higher in men, the mean value of LBT was higher in women. A significant difference was observed between the mean values of SF depth and BBT in the left jaw according to the gender of the individuals (p<0.05). The SF depth and BBT values of males were higher than those of females. According to the gender of the individuals, the distribution of the data related to the tooth alignment of the deepest point of the SF in the left jaw showed a significant difference (p<0.05). The rate of 'undeterminate' ones in the left jaw was found to be lower in males than in females. According to the gender of the individuals, the distribution of the data related to the presence of the 2nd molar tooth in the left jaw in the mouth showed a significant difference (p<0.05). The rate of the presence of the 2nd molar tooth in the left jaw in the mouth in males was higher than the rate of the presence of the 2nd molar tooth in the left jaw in the mouth in females.

The comparison of the findings regarding the anatomical structure of the right and left jaws and the dental regions examined according to the age of the individuals is shown in Table **2**.

According to the age of the individuals, the distribution of the data on the tooth alignment of the region where the SF was deepest in the right and left jaws showed a significant difference (p<0.05). In both the right and left jaws, especially with the increase in the age of the individuals, in the distribution of the data related to the tooth alignment of the region where the SF was deepest, depending on the 'edentulous' rates, the rate of 'undetermined' increased. The distribution of the presence of 1st and 2nd molars in the right and left jaws according to the age of the individuals showed a significant difference (p<0.05). In both jaws, there was a decrease in the presence of 1st and 2nd molars with increasing age. The distribution of the data on the relationship between the vertical position of the MC in the left jaw and SF according to the age of the individuals showed a significant difference (p<0.05). As the age of the individuals increased, there was an increase in parallel data and a decrease in inferior data in the relationship between the vertical position of the MC and SF.

The comparison of the findings related to the BBT, LBT, MC, and the examined tooth regions in the right jaw according to the SF depth types of the individuals is shown in Table **3**.

A significant difference was observed between the mean values of BBT and LBT in both right and left jaws according to the SF depth types of the individuals (p<0.05). According to the results, as the SF depth types of the individuals increased in both jaws, BBT and LBT values decreased. According to the SF depth types of the individuals, the distribution of the data related to the presence of the 2nd molar tooth in the left jaw in the mouth showed a significant difference (p<0.05). The rate of the 2nd molar tooth in the mouth in individuals with SF depth type I was lower than the rate of the 2nd molar tooth in the mouth in individuals with SF depth type II and type III.

The comparison of the findings regarding the anatomical structure of the right jaw and the examined tooth regions according to the presence of teeth is shown in Table **4**.

There was a significant difference between the distributions of SF depth types in the right jaw, SF depth, and BBT averages according to the dentition status of the individuals (p<0.05). The SF Type III ratios, SF depths, and BBT mean values of edentulous individuals were lower than those of toothed individuals. There was a significant difference between the mean values of LBT in the left jaw according to the dentition status of the individuals (p<0.05). The mean LBT values of individuals with teeth in the left jaw were lower than those of individuals with a single tooth.

In our study, when the mean values of SF depth, BBT, and LBT in the right and left jaws were compared, it was observed that the mean values were similar and there was no significant difference between the values (p>0.05).

## 4 DISCUSSION

Located on the lingual side of the posterior part of the mandible, the SF is a concavity where part of the submandibular gland is located. Perforation of the lingual cortex in the region of the fossa during implant placement in the mandibular posterior region brings various complications. The risk of perforation of the lingual cortex increases as the depth of the SF increases [6, 21].

Previous studies have used imaging modalities such as periapical, panoramic, CT, and CBCT in the evaluation of SF. The main disadvantages of periapical and panoramic images in image evaluation are that they cannot provide sufficient information about the depth of the bone due to the 2-dimensional nature of the images. For this reason, cross-sectional images of the region should be examined and analyzed in detail before surgery to place the implants in the appropriate size and position and to adjust the bucco-lingual angles as accurately as possible [1, 6, 13, 20]. CBCT provides high-quality images at a low radiation dose, allowing for highly accurate three-dimensional image analysis of the region to be examined. For this reason, CBCT is considered the gold standard for the evaluation of alveolar bone [9, 22].

In one study, the incidence of lingual plate perforation was determined to be between 1.1% and 1.2%, and palpation of the lingual posterior region of the mandible was recommended to detect the presence of a lingual cavity before surgery [21]. In other studies, the rate of lingual concavity was determined as 36-39% [23] and 60% [24].

In their study, Parnia *et al*. [6] stated that complications during surgical procedures would increase in patients with a fossa depth greater than 2 mm. In the Yıldız *et al*. [25] study, when the fossa depth is between 1.1 mm and 4.6 mm, a lingual implant angulation between 84° and 62° can be planned to prevent damage to vital structures.

Parnia *et al*. [6] retrospectively evaluated 100 spiral CT images selected from patients with partially edentulous mandibles. They found that 20% of the cases were type I, 52% were type II, and 28% were type III, and SF depth was ≥2 mm in 80% of the jaws. In a study by Yildiz *et al*. [25] evaluating spiral CT images of 154 dry adult human hemimandibula, the SF depth was ≥2 mm in 71.5% of the hemimandibula. In our study, similar to these results, SF depth was ≥2 mm in 71.4% of the images analyzed in the right jaw and 75.4% in the left jaw. In most of the patients evaluated in our study, the SF depth of more than 2 mm. This should be taken into consideration in terms of lingual plate perforation in surgical procedures to be performed in the relevant region.

Similar to our study, Ramaswamy *et al*. [13] evaluated the SF depth separately in the right and left hemispheres of the jaw, and similar to our study, they found that the SF depth was higher in men than in women.

In contrast to our findings, Bayrak *et al*. [20], Borahan *et al*. [4], Sumer *et al*. [2], Farahani *et al*. [26], Panjnoush *et al*. [27], and İçöz *et al*. [1] recorded the fossa depth as type I > type II > type III in their studies on SF depth. The reasons why these results are different from the results obtained in our study may be the differences in the dental status of the individuals included in the study.

In the results of many studies evaluating the distribution of SF depth according to gender in the literature, it was reported that SF depth was higher in men than in women [1, 6, 13, 26, 27]. Similar results were obtained in our study. The fact that men have coarser and sharper lines than women in terms of skeletal structure may affect the results of our study. In addition, the higher amount of resorption in the alveolar crest in postmenopausal women may also have affected these results.

Similar to our study, Borahan *et al*. [4] found that the right and left Type 1 SF depth in women (57.7%) was higher than in men (38.6%) and stated that this result may be related to hormonal conditions. In a study by Yildiz *et al*. [25], they found a statistically significant difference between the right and left sides of the mandibles in terms of SF depth, but emphasized that this difference was clinically insignificant.

Bayrak *et al*. [20]; İçöz *et al*. [1]; de Souza *et al*. [9]; Bayrakdar *et al*. [28]; Kamburoğlu *et al*. [5]; Parnia *et al*. [6] and Borahan *et al*. [4] did not find a significant difference in SF depth according to age groups, similar to our study. They attributed this to the lack of severe resorption in most of the patients.

In a study conducted by Bayrakdar *et al*. [28], the rate of concavity was found to be statistically significant and higher in the toothed regions than in the edentulous regions. The results of our study also support this result. The reason for the lower SF depth in edentulous individuals may be the crest resorption and atrophy due to the disuse of the edentulous region. In contrast to our study, Nilsun *et al*. [18] found that the depth of concavity was higher in edentulous patients than in toothed patients. They stated that this was due to the higher tendency for bone loss in the height and width of the lower jaw in edentulous patients.

Similar to our study, İçöz *et al*. [1] and Bayrak *et al*. [20] found that BBT and LBT decreased as the SF depth increased. In their study, Bayrak *et al*. [20] stated that an increase in SF depth would mean that the mandible would be narrower and the distance from the buccal and lingual cortical surfaces to the MC would decrease.

Bayrak *et al*. [20] found that BBT values were significantly lower on the left side than on the right side. They found that LBT values were lower on the right side than on the left side. Kawashima *et al*. [29] did not find a significant difference between the jaws when LBT on the right and left sides were compared. In our study, as the BBT values in the right jaws of the individuals increased, a decrease was observed in the LBT values, while no relationship was found between the BBT and LBT in the left jaws.

In their study, İçöz *et al*. [1] found that LBT values were higher in females than in males. Bayrak *et al*. [20] found that BBT values were higher in male individuals than in female individuals. They stated that this may be because women have shorter height and softer jaw lines. When they evaluated LBT values according to gender, they saw a difference between genders, but they did not find this difference statistically significant Bayrak *et al*. [20]. In our study, SF depth and BBT values were found to be higher in males than females in the right and left jaws according to the gender of the individuals. LBT values were found to be higher in women in the right jaw, while no significant difference was observed in the left jaw. The MC position may have had an effect on the BBT value being higher in male individuals than in female individuals. If the MC is more in the vestibule in males, this may cause BBT to be higher and LBT to be lower in males than in females. de Oliveira *et al*. [30] found that BBT and LBT values were lower in females than males, but the differences were not statistically significant. Kawashima *et al*. [29] found that BBT values were significantly lower on the right side than on the left side in both men and women. When they evaluated LBT values, they found that LBT values on the left side were significantly higher in women than in men. The researchers stated that this may be because the MC is located more superiorly in women than in men.

Similar to our study, İçöz *et al*. [1] did not find a statistically significant difference between the age of individuals and BBT and LBT in their study. Oliveira *et al* [30] found that BBT and LBT values were lower in the elderly than in the young, but they did not find the differences statistically significant. This result may be because the same number of images was not evaluated in each age group.

de Souza *et al*. [9] found that there was a significant negative correlation between bone thickness and patient age in the mandible, regardless of the jaw direction evaluated. de Souza *et al*. [9] attributed this to bone loss in the alveolar bone with aging. The difference between our study and the study of de Souza *et al*. [9] may be due to the different methods used to measure and evaluate bone thickness.

Katranji *et al*. [31] in a study on a total of 28 cadavers, including both toothed and edentulous cadavers and found that BBT was higher in edentulous cadavers, while LBT was higher in toothed patients. In our study, unlike these results, there was no significant difference between the mean values of LBT in the right jaw according to the dentition status of the individuals; however, a significant difference was observed between the mean values of BBT. In the right jaw, the BBT of edentulous individuals was found to be lower than that of single-toothed and toothed individuals. In the left jaw, while there was no significant difference between the mean values of BBT according to the dentition status of the individuals, the LBT of the individuals with teeth was found to be lower than that of the individuals with single teeth. In addition, a low-level negative correlation was found between the presence of 1st and 2nd molars and LBT in the left jaw. The presence of 1st and 2nd molars in the left jaw decreased the mean values of LBT. Since the LBT value was measured higher in patients with a single tooth in the left jaw than in patients with two teeth, a careful evaluation should be made in order to prevent lingual perforation in patients who have lost one of their first and second molars before surgical procedures are performed in the relevant region. The reason why the results of the two studies were not compatible may be that the measurement method was different from our study because the study was cadaveric.

In their study, Bayrak *et al*. [20] stated that the region with the highest SF depth in the right and left jaw was the second molar region, and the region with the lowest SF depth was the second premolar region. Nilsun *et al*. [18] examined the patients in four different groups according to the Kennedy classification, according to dentition status. They stated that the tooth groups with the deepest concavity of the SF were the 1st and 2nd molars for all Kennedy Class groups. de Souza *et al*. [9] determined the mandibular molar region as the region with the highest SF depth. In our study, similar to these studies, the tooth regions with the deepest SF in the right and left jaws were determined as the first and second molar regions. In a small number of cases in our study, the tooth regions with the deepest SF were determined as the third molar and the second premolar.

In the study by Parnia *et al*. [6], the MC was found to be inferior to the SF in all patients participating in the study. Similar to the results of our study, Farahani *et al*. [26] found that the relationship between the vertical position of the MC and the deepest part of the SF was mostly inferior. This was followed by a parallel and superior relationship. The reason for this difference between the studies may be the different positioning of the MC within the bone. In their study, Felice *et al* [32] stated that the use of 7 mm implants would reduce the risk in cases where the residual bone height on the MC is between 7 and 8 mm.

Farahani *et al*. [26] found no significant difference between genders in the relationship between the vertical position of the MC and the deepest region of the SF. They also found that the vertical position of the MC was located inferior to the SF in both sexes, and there was no significant difference between the sexes. In the study of Ramaswamy *et al*. [13], parallel relationship was most commonly seen in females, while an inferior relationship was most commonly seen in males. In our study, the distributions of the data regarding the vertical position of the MC in the right and left jaw and the region where the SF is deepest relative to each other did not show a significant difference according to the gender of the individuals. The reason why the results of the study of Ramaswamy *et al*. [13] differed from the results of our study may be that the number of men and women was not equal in our study.

In our study, the distribution of the data related to the relationship between the vertical position of the MC in the right jaw and the deepest region of the SF according to the age of the individuals did not show a significant difference. In the left jaw, as the age of the individuals increased, the proportion of parallel ones increased and the proportion of inferior ones decreased in the relationship between the vertical position of the MC with the deepest region of the SF.

In contrast to our findings, Bayrak *et al*. [20] examined the position of the vertical position of the MC relative to the SF in panoramic images and concluded that the SF was deeper in patients with the canal in the inferior position. The researchers also stated that three-dimensional images are needed for more detailed examination. The difference in the study results may have resulted from the difference in the imaging method used in the study. In our study, a detailed examination was performed on cross-sectional sections by examining KIBT images, while Bayrak *et al*. [20] evaluated their study on panoramic images.

There were also various limitations in our study. Examples of these limitations include the unequal number of male and female individuals in the individuals whose images were evaluated, the fact that an equal number of patients were not included in each age group when the distribution of the individuals whose images were evaluated according to age groups was examined, the fact that clinical examinations of the patients were not performed due to the retrospective study, and the fact that information on when the edentulous patients had their teeth extracted and how long they had been edentulous could not be obtained because clinical examinations were not performed in the patients when examining the data according to dentition status.

The lower number of male individuals in our study may have affected the higher SF and BCR values in males. The fact that the number of toothed individuals was significantly higher than edentulous individuals in our study may have influenced the higher SF and BBT values in edentulous individuals. In addition, knowing the duration of edentulous patients' edentulousness might have helped us to understand the effect of atrophy on SF, BBT, and LBT values. Since the study was conducted on only one population, it is recommended to conduct studies on different populations in order to make generalizations.

## CONCLUSION

The results obtained from our study are as follows:

In the majority of the patients evaluated in our study, the SF depth was found to be more than 2 mm. Since this situation increases the risk of complications, the relevant region should be examined in detail before surgical procedures are performed in the mandibular posterior region. SF depth and BBT values in the right and left jaws were higher in men than in women. While LBT was found to be higher in females in the right jaw, no significant difference was found between the sexes in the left jaw. The deepest tooth region of the SF was found at the 2nd molar tooth level in both men and women. When the relationship between the vertical position of the MC and the region where the SF was deepest was examined, it was observed that the MC was in the inferior position in most of the patients. As the depth of the SF increased in the patients, BBT and LBT values decreased. When the depth of the SF was analyzed according to the dentition status in the right and left jaws, it was found that the depth of the SF in the right jaw was higher than the depth of the SF in the edentulous patients, while no significant difference was found between the depth of the SF and the tooth status in the left jaw.

SF is a rich and risky region in terms of anatomical formations. As the depth of the SF increases, the risk of complications increases. Sublingual artery injuries that may occur during procedures in the region may lead to risky hemorrhages. Excessive hemorrhage may lead to serious life-threatening problems due to obstruction of the upper airway. As a result of mandibular canal damage, paresthesia may occur in the region. To prevent these complications, the region should be examined, and detailed information about the anatomy of the region should be obtained before surgical procedures. Before performing implant surgery in the mandibular posterior region, CBCT images should be examined first to prevent possible complications. When cross-sectional images are examined at 1 mm intervals, more detailed information about the relevant region will be obtained. In patients with an SF depth of more than 2 mm, implants should be placed at an angle so as not to cause perforation in the lingual cavity. When the results of our study are evaluated, a detailed analysis of the region with CBCT before surgical interventions in the region is recommended to reduce the risk of complications.

## Figures and Tables

**Table 1:** Comparison of the findings regarding the anatomical structure of the right and left jaw and the examined dental regions according to the gender of the ındividuals.

**The Region where the Teeth are Located and the Anatomical Structures**		**Right Half Jaw**			**Left Half Jaw**		
		**Male (n:86)**	**Female (n:134)**	-	**Male (n:86)**	**Female (n:134)**	-
		**Number (%)**	**Number (%)**	**p**	**Number (%)**	**Number (%)**	**p**
SF depth	Type I	14 (16,3)	49 (36,6)	0,000**	13 (15,1)	41 (30,6)	0,001**
	Type II	41 (47,7)	67 (50,0)		41 (47,7)	71 (53,0)	
	Type III	31 (36,0)	18 (13,4)		32 (37,2)	22 (16,4)	
The tooth area where the SF is deepest	1. Premolar	**-**	**-**	0,263	1 (1,2)	2 (1,5)	0,007**
	1. Molar	20 (23,3)	33 (24,6)		23 (26,7)	42 (31,3)	
	2. Molar	48 (55,8)	65 (48,5)		48 (55,8)	54 (40,3)	
	3. Molar	8 (9,3)	8 (6,0)		8 (9,3)	5 (3,7)	
	Undetermined	10 (11,6)	28 (20,9)		6 (7,0)	31 (23,1)	
Relationship between the vertical position of the MC and SF	Superior	2 (2,3)	4 (3,0)	0,721	2 (2,3)	1 (0,7)	0,381
	Paralel	36 (41,9)	49 (36,6)		43 (50,0)	59 (44,0)	
	İnferior	48 (55,8)	81 (60,4)		41 (47,7)	74 (55,2)	
1. Molar	Unlocated	24 (27,9)	49 (36,6)	0,236	22 (25,6)	46 (34,3)	0,222
	Located	62 (72,1)	85 (63,4)		64 (74,4)	88 (65,7)	
2. Molar	Unlocated	20 (23,3)	30 (22,4)	1,000	9 (10,5)	33 (24,6)	0,015*
	Located	66 (76,7)	104 (77,6)		77 (89,5)	101 (75,4)	
		Mean± S.D.	Mean± S.D.	p	Mean± S.D.	Mean± S.D.	P
SF depth^t^		2,77 ± 0,76	2,44 ± 0,73	0,000**	2,75 ± 0,73	2,29 ± 0,59	0,000**
BBT^t^		5,81 ± 1,38	5,52 ± 1,34	0,013*	5,79 ± 1,29	5,34 ± 1,33	0,012*
LBT^t^		2,11 ± 0,81	2,28 ± 0,8	0,000**	2,12 ± 0,84	2,34 ± 0,91	0,074

**Note:** *p<0,05; **p<0,01, Categorical data: Chi-Square Analysis; t: Independent Sample T Test.

**Table 2:** Comparison of the anatomical structure of the right and left jaw and the findings related to the examined tooth regions according to the age of the ındividuals.

		**Right Half Jaw**					**Left Half Jaw**				
**Tooth Alignment and Anatomical Structures**		**19-29 years (n:87)**	**30-39 years (n:61)**	**40-54 years (n:39)**	**55 years and above (n:33)**	-	**19-29 years (n:87)**	**30-39 years (n:61)**	**40-54 years (n:39)**	**55 years and above (n:33)**	-
		**Number (%)**	**Number (%)**	**Number (%)**	**Number (%)**	**p**	**Number (%)**	**Number (%)**	**Number (%)**	**Number (%)**	**p**
SF depth	Type I	21 (24,10)	19 (31,10)	10 (25,60)	13 (39,40)	0,608	14 (16,10)	19 (31,10)	10 (25,60)	11 (33,30)	0,310
	Type II	44 (50,60)	31 (50,80)	21 (53,80)	12 (36,40)		47 (54,00)	30 (49,20)	19 (48,70)	16 (48,50)	
	Type III	22 (25,30)	11 (18,00)	8 (20,50)	8 (24,20)		26 (29,90)	12 (19,70)	10 (25,60)	6 (18,20)	
The tooth area where the SF is deepest	1. Premolar	-	-	-	-	0,000**	1 (1,10)	2 (3,30)	0 (0,00)	0 (0,00)	0,000**
	1. Molar	29 (33,30)	16 (26,20)	5 (12,80)	3 (9,10)		33 (37,90)	21 (34,40)	7 (17,90)	4 (12,10)	
	2. Molar	47 (54,00)	38 (62,30)	21 (53,80)	7 (21,20)		46 (52,90)	25 (41,00)	23 (59,00)	8 (24,20)	
	3. Molar	9 (10,30)	2 (3,30)	4 (10,30)	1 (3,00)		6 (6,90)	6 (9,80)	1 (2,60)	0 (0,00)	
	Undetermined	2 (2,30)	5 (8,20)	9 (23,10)	22 (66,70)		1 (1,10)	7 (11,50)	8 (20,50)	21 (63,60)	
Relationship between the vertical position of the MC and SF	Superior	1 (1,10)	0 (0,00)	3 (7,70)	2 (6,10)	0,140	1 (1,10)	0 (0,00)	2 (5,10)	0 (0,00)	0,003**
	Paralel	32 (36,80)	23 (37,70)	14 (35,90)	16 (48,50)		33 (37,90)	26 (42,60)	18 (46,20)	25 (75,80)	
	İnferior	54 (62,10)	38 (62,30)	22 (56,40)	15 (45,50)		53 (60,90)	35 (57,40)	19 (48,70)	8 (24,20)	
1. Molar	Unlocated	14 (16,10)	15 (24,60)	17 (43,60)	27 (81,80)	0,000**	10 (11,50)	11 (18,00)	19 (48,70)	28 (84,80)	0,000**
	Located	73 (83,90)	46 (75,40)	22 (56,40)	6 (18,20)		77 (88,50)	50 (82,00)	20 (51,30)	5 (15,20)	
2. Molar	Unlocated	5 (5,70)	9 (14,80)	10 (25,60)	26 (78,80)	0,000**	2 (2,30)	10 (16,40)	9 (23,10)	21 (63,60)	0,000**
	Located	82 (94,30)	52 (85,20)	29 (74,40)	7 (21,20)		85 (97,70)	51 (83,60)	30 (76,90)	12 (36,40)	
		Mean± S.D.	Mean± S.D.	Mean± S.D.	Mean± S.D.	p	Mean± S.D.	Mean± S.D.	Mean± S.D.	Mean± S.D.	p
SF depth^F^		2,50 ± 0,70	2,41 ± 0,73	2,41 ± 0,69	2,33 ± 0,82	0,683	2,55 ± 0,68	2,41 ± 0,68	2,51 ± 0,68	2,30 ± 0,69	0,306
BBT^F^		5,52 ± 1,27	5,77 ± 1,45	5,17 ± 1,43	5,47 ± 1,17	0,186	5,47 ± 1,19	5,63 ± 1,48	5,48 ± 1,37	5,48 ± 1,38	0,896
LBT^F^		2,19 ± 0,74	2,37 ± 0,82	2,34 ± 1	2,33 ± 0,86	0,565	2,21 ± 0,85	2,18 ± 0,89	2,29 ± 0,89	2,47 ± 0,95	0,469

**Note:** *p<0,05; **p<0,01, Categorical data: Chi-Square Analysis; F: One Way ANOVA Test.

**Table 3:** Comparison of the findings regarding the BBT, LBT, MC, and examined tooth regions in the right and left jaws according to the SF Depth types of individuals.

		**Right Half Jaw**				**Left Half Jaw**			
**Tooth Regions and Anatomical Structures**		**Type I (n:63)**	**Type II (n:108)**	**Type III (n:49)**	-	**Type I (n:63)**	**Type II (n:108)**	**Type III (n:49)**	-
		**Number (%)**	**Number (%)**	**Number (%)**	**p**	**Number (%)**	**Number (%)**	**Number (%)**	**p**
The tooth area where the SF is deepest	1. Premolar	-	-	-	0,150	0 (0,0)	1 (0,9)	2 (3,7)	0,123
	1. Molar	17 (27,0)	27 (25,0)	9 (18,4)		11 (20,4)	38 (33,9)	16 (29,6)	
	2. Molar	24 (38,1)	58 (53,7)	31 (63,3)		27 (50,0)	50 (44,6)	25 (46,3)	
	3. Molar	6 (9,5)	6 (5,6)	4 (8,2)		1 (1,9)	8 (7,1)	4 (7,4)	
	Undetermined	16 (25,4)	17 (15,7)	5 (10,2)		15 (27,8)	15 (13,4)	7 (13,0)	
Relationship between the vertical position of the MC and SF	Superior	2 (3,2)	2 (1,9)	2 (4,1)	0,179	1 (1,9)	0 (0,0)	2 (3,7)	0,198
	Paralel	29 (46,0)	44 (40,7)	12 (24,5)		29 (53,7)	52 (46,4)	21 (38,9)	
	İnferior	32 (50,8)	62 (57,4)	35 (71,4)		24 (44,4)	60 (53,6)	31 (57,4)	
1. Molar	Unlocated	28 (44,4)	33 (30,6)	12 (24,5)	0,061	23 (42,6)	31 (27,7)	14 (25,9)	0,099
	Located	35 (55,6)	75 (69,4)	37 (75,5)		31 (57,4)	81 (72,3)	40 (74,1)	
2. Molar	Unlocated	20 (31,7)	22 (20,4)	8 (16,3)	0,111	17 (31,5)	16 (14,3)	9 (16,7)	0,027*
	Located	43 (68,3)	86 (79,6)	41 (83,7)		37 (68,5)	96 (85,7)	45 (83,3)	
		Mean± S.D.	Mean± S.D.	Mean± S.D.	p	Mean± S.D.	Mean± S.D.	Mean± S.D.	P
SF depth^F^		1,62 ± 0,22	2,45 ± 0,29	3,45 ± 0,47	0,000**	1,65 ± 0,24	2,40 ± 0,28	3,42 ± 0,34	0,000**
BBT^F^		6,01 ± 1,33	5,43 ± 1,41	5,10 ± 1,03	0,004**	5,98 ± 1,31	5,53 ± 1,31	5,03 ± 1,24	0,001**
LBT^F^		2,57 ± 0,83	2,31 ± 0,77	1,86 ± 0,81	0,003**	2,75 ± 1,02	2,22 ± 0,78	1,84 ± 0,7	0,000**

**Note:** *p<0,05; **p<0,01, Categorical data: Chi-Square Analysis; F: One Way ANOVA Test

**Table 4:** Comparison of the anatomical structure of the right and left jaw and the findings related to the examined dental regions according to the presence of teeth of the ındividuals.

-	**Right Half Jaw**				**Left Half Jaw**			
**Tooth Regions and Anatomical Structures**	**Absence (n:38)**	**Mono tooth (n:47)**	**Two teeth (n:135)**	-	**Absence (n:38))**	**Mono tooth (n:47)**	**Two teeth (n:135)**	-
	**Number (%)**	**Number (%)**	**Number (%)**	**p**	**Number (%)**	**Number (%)**	**Number (%)**	**p**
SF depth	Type I	16 (42,1)	16 (34,0)	31 (23,0)	0,000**	15 (40,5)	10 (27,8)	29 (19,7)	0,125
	Type II	17 (44,7)	21 (44,7)	70 (51,9)		15 (40,5)	17 (47,2)	80 (54,4)	
	Type III	5 (13,2)	10 (21,3)	34 (25,2)		7 (18,9)	9 (25,0)	38 (25,9)	
Relationship between the vertical position of the MC and SF	Superior	1 (2,6)	2 (4,3)	3 (2,2)	0,272	1 (2,7)	0 (0,0)	2 (1,4)	0,220
	Paralel	20 (52,6)	19 (40,4)	46 (34,1)		22 (59,5)	19 (52,8)	61 (41,5)	
	İnferior	17 (44,7)	26 (55,3)	86 (63,7)		14 (37,8)	17 (47,2)	84 (57,1)	
		Mean± S.D.	Mean± S.D.	Mean± S.D.	p	Mean± S.D.	Mean± S.D.	Mean± S.D.	P
SF depth^F^		2,17 ± 0,64	2,43 ± 0,8	2,51 ± 0,7	0,032**	2,29 ± 0,65	2,41 ± 0,8	2,53 ± 0,66	0,135
BBT^F^		4,97 ± 1,22	5,82 ± 1,26	5,57 ± 1,37	0,011*	5,06 ± 1,37	5,54 ± 1,41	5,62 ± 1,28	0,066
LBT^F^		2,32 ± 0,8	2,39 ± 0,72	2,24 ± 0,87	0,534	2,47 ± 0,81	2,54 ± 1,04	2,13 ± 0,84	0,011*

**Note:** *p<0,05; **p<0,01, Categorical data: Chi-Square Analysis; F: One Way ANOVA Test.

## Data Availability

All data generated or analyzed during this study are included in this published article.

## LIST OF ABBREVIATIONS

SF: Submandibular Fossa
CBCT: Cone Beam Computed Tomography
BBT: Buccal Bone Thickness
LBT: Lingual Bone Thickness
MC: Mandibular Canal
CT: Computed Tomography

## STANDARDS OF REPORTING

STROBE guidelines were followed.
